# The role of osteopontin in the progression of solid organ tumour

**DOI:** 10.1038/s41419-018-0391-6

**Published:** 2018-03-02

**Authors:** Hailin Zhao, Qian Chen, Azeem Alam, Jiang Cui, Ka Chun Suen, Aurelie Pac Soo, Shiori Eguchi, Jianteng Gu, Daqing Ma

**Affiliations:** 1 Department of Surgery and Cancer, Faculty of Medicine, Anaesthetics, Pain Medicine and Intensive Care, Imperial College London, Chelsea and Westminster Hospital, London, UK; 2 Department of Anaesthesiology, Southwest Hospital, Third Military Medical University, Chongqing, China

## Abstract

Osteopontin (OPN) is a bone sialoprotein involved in osteoclast attachment to mineralised bone matrix, as well as being a bone matrix protein, OPN is also a versatile protein that acts on various receptors which are associated with different signalling pathways implicated in cancer. OPN mediates various biological events involving the immune system and the vascular system; the protein plays a role in processes such as immune response, cell adhesion and migration, and tumorigenesis. This review discusses the potential role of OPN in tumour cell proliferation, angiogenesis and metastasis, as well as the molecular mechanisms involved in these processes in different cancers, including brain, lung, kidney, liver, bladder, breast, oesophageal, gastric, colon, pancreatic, prostate and ovarian cancers. The understanding of OPN’s role in tumour development and progression could potentially influence cancer therapy and contribute to the development of novel anti-tumour treatments.

## Facts

- Osteopontin (OPN) is a versatile protein that acts on various receptors which are associated with different signalling pathways implicated in cancer.
- OPN mediates critical processes for cancer progression such as immune response, cell adhesion and migration, and tumorigenesis.

## Open questions

- What are the precise molecular mechanisms of osteopontin in tumour progression?
- How is osteopontin related to the diagnosis and prognosis of cancer?
- Which therapeutic strategy targeting osteopontin would be the most effective in treating cancer in the clinical settings?

## Introduction

Malignant cancers are described as uncontrolled growths which overcome replicative senescence, resulting in metastatic disease^1^. There are many factors that have essential functions in the regulation of the survival, proliferation, adhesion and migration of neoplastic cells, such as various growth factors and cytokines^1^. Recently there has been ongoing research regarding the role of osteopontin in tumour progression. This review provides an insight into the potential role that osteopontin may play in tumour cell proliferation, angiogenesis and metastasis and the molecular mechanisms underlying tumour progression in various organs.

### Biology of osteopontin

#### Molecular pathway of osteopontin

**Fig. 1:**
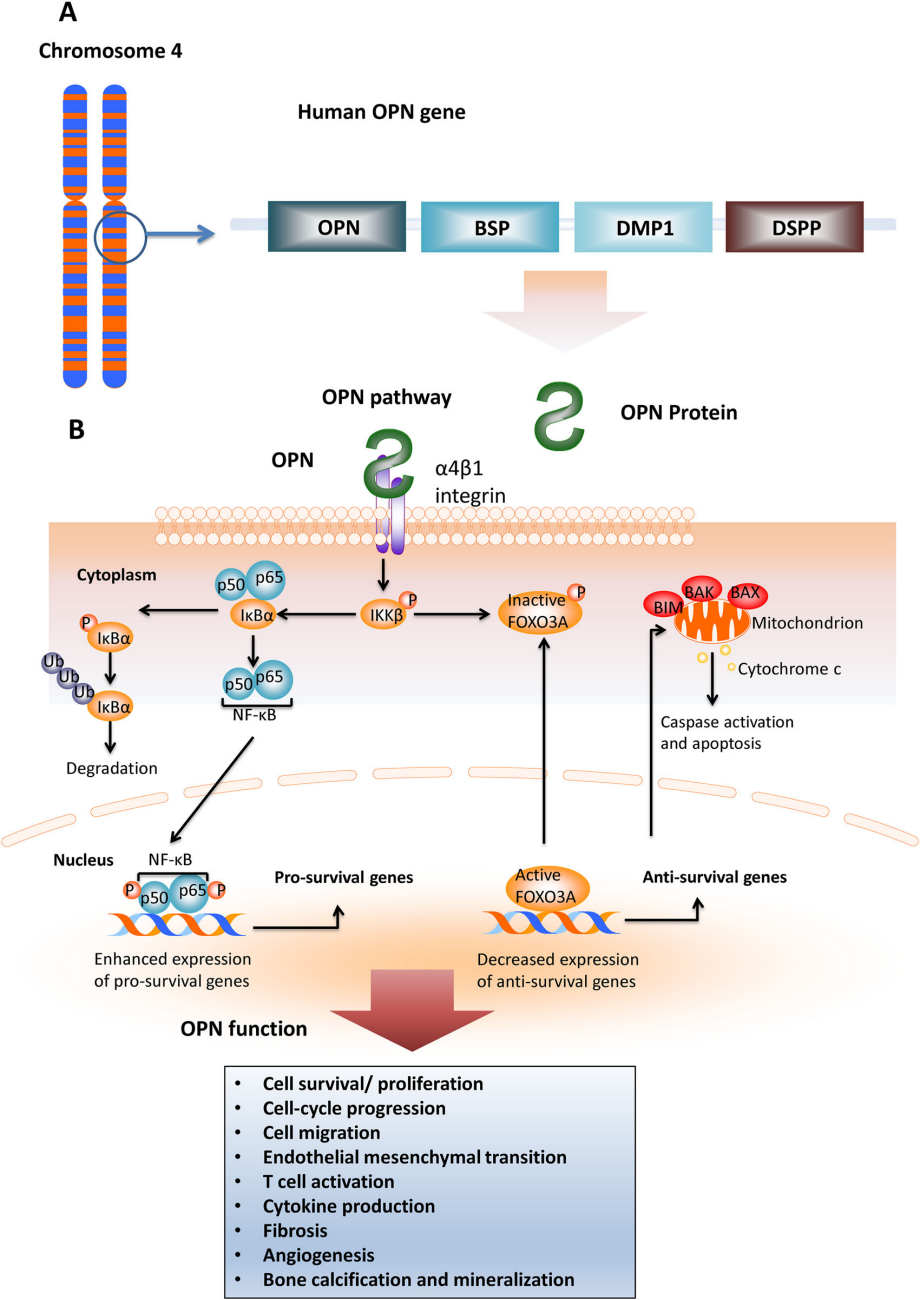
The OPN gene, protein, signalling pathway and function in normal tissues. **a** The schematic representation of the location of OPN on human chromosome 4.OPN is at location 4q22.1. Genes encompassed within a 600 kb region on chromosome 4 encodes several noncollagenous bone and dentin proteins including OPN, bone sialoprotein(BSP), dentin matrix protein I (DMPI) and dentin sialophosphoportin (DSPP). All of them are classified as small intergrin-binding ligand N-linked glycoprotein (SIBLING) family proteins. **b** The signalling pathway of osteopontin. Osteopontin binds integrin α4β1 which causes degradation of phospharylated inhibitor of nuclear factor kappa-B kinase subunit beta (IKKβ). Inhibitor of nuclear transcription factor kappa-B (IκBα) and nuclear transcription factor kappa-B (NF-κB; p50 and p65) are both freed following this process. IκBα is degraded by the ubiquitination pathway, while NF-κB enters the cell nucleus where it is phosphorylated and it enhances the expression of pro-survival genes. Moreover, upon binding of osteopontin to integrin α4β1, phosphorylated IKKβ causes inactivation of Forkhead box O3 (FOXO3A). Active FOXO3A is important in decreasing the expression of anti-survival genes such as BIM, BAK and BAX which cause caspase activation and cell apoptosis via mitochondrion and release of cytochrome c. Activation of OPN mediate a diverse range of cellular function, including cell survival/ proliferation, cell-cycle progression, cell migration, endothelial mesenchymal transition, T cell activation, cytokine production, fibrosis, angiogenesis and bone calcification and mineralisation^2^

Not only is Osteopontin (OPN) a major non-collagenous bone matrix protein (which gave it its name), it also plays a role in other systems. For example, as an intrinsic component of the immune system, OPN controls cytokine production and regulates cell trafficking ^2^. OPN is an acidic arginine-glycine-aspartate-containing adhesive glycoprotein with a molecular mass of approximately 44 kDa (Fig. 1)^2^. The central section of the molecule consists of sequences that interact with seven integrins, such as *α*v*β*3 and *β*5, as well as a sequence of *β*1-consisting integrins and a cryptic *α*9*β*1 region that is only functional after protease cleavage, thus suggesting a specific role for OPN fragments^3^. Not only does OPN communicate with cells via integrins, but also via CD44, which involves an intracellular interaction^4^. OPN has a conserved region that separates both the integrin and CD44 binding domains, which possess distinct signalling functions^5^. Different isoforms of CD44 are produced by alternate splicing of the pre-mRNA and have been found in different cancer cells^6^. The presence of these isoforms in different cancers suggests that they can be used as a marker for cancer progression and patient survival.

OPN is mainly synthesised by osteoblasts, osteocytes and other hematopoietic cells^7^. In addition, OPN is also secreted by neutrophils, dendritic cells, NK cells, T cells and B cells^8^. The structure of OPN is relatively simple, with about 300 amino acids composing a single chain polypeptide and expressed as a 33-KDa nascent protein^8^. OPN is actively involved in many physiological functions. It is an important factor in bone remodelling, anchoring osteoclasts to the mineral matrix of bones^9^. In addition, OPN regulates both the innate and adaptive immune systems. Functioning in a similar manner to T cell helper 1 cytokines, OPN promotes a cell-mediated immune response^10^. OPN contains an integrin –binding RGD sequence that interacts with CD44v 6/7, which is a cell surface glycoprotein involved in cell-cell interactions. OPN can also activate intracellular pathways to regulate gene expression within the immune system^11^. The important role of OPN in cell differentiation has been thoroughly investigated and it has been shown to suppress adipogenic differentiation and promote osteogenic differentiation in mesenchymal stem cells by interacting with integrin αvβ1^12^. In addition, OPN regulates cytokine production ^2^. In summary, OPN is a protein with a diverse array of functions, ranging from the regulation of bone mineralisation, to recruiting macrophages and facilitating cell adhesion and migration.

### General role of osteopontin in cancer progression

**Fig. 2:**
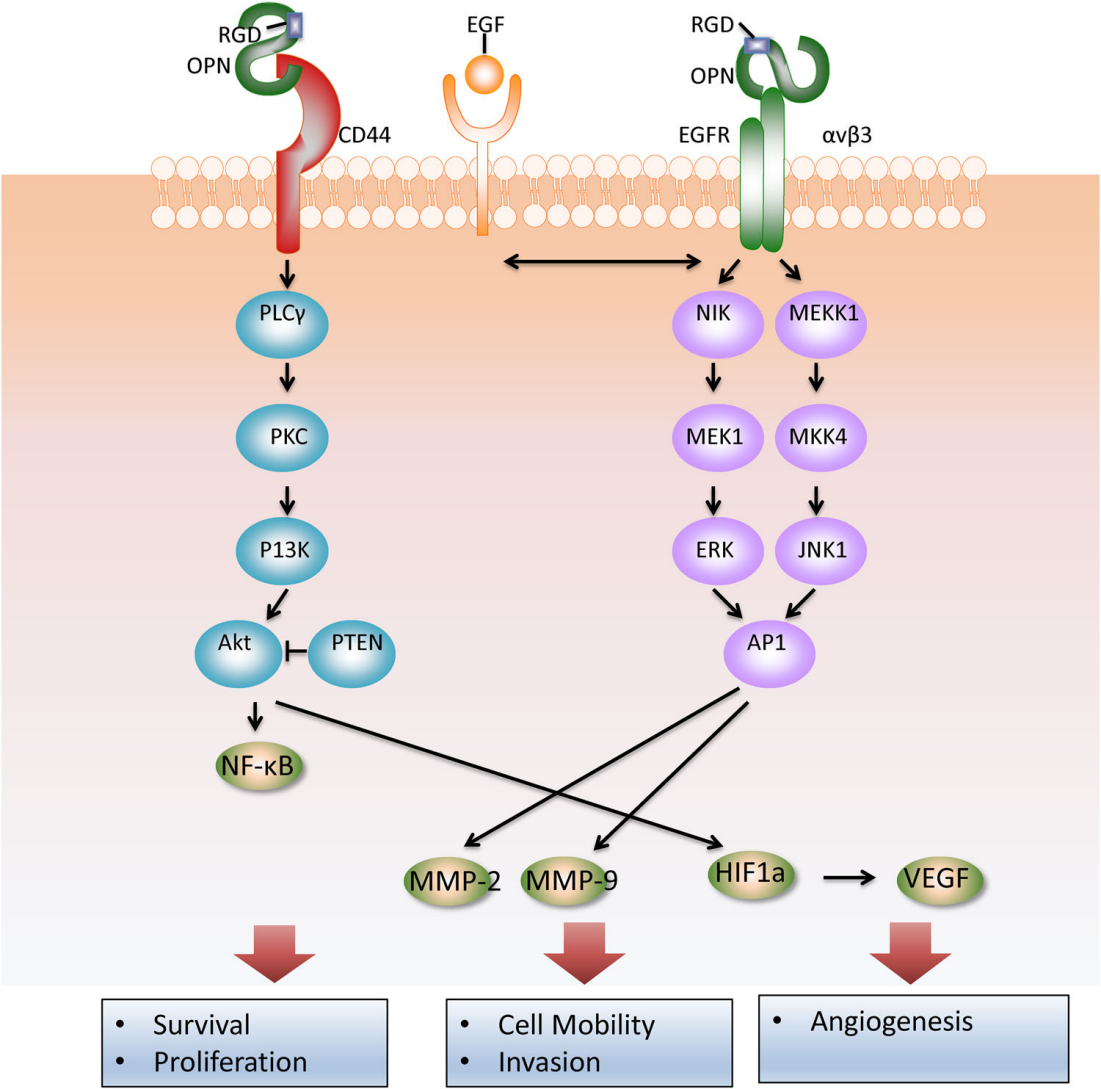
The signalling pathway of osteopontin in tumour progression. Osteopontin (OPN) can interact with several integrins in Arg-Gly-Asp (RGD) dependent and RGD independent manners^13^. OPN can also interact with the CD44 family of receptors. Upon binding of receptors, OPN can induce cellular reactions include: survival, motility and tumour progression, MMP localisation and complement inhibition. By interacting with CD44 family of receptors, OPN can activate the cell anti-apoptotic signals in tumour cells through hospholipase C-γ(PLCγ–protein kinase C (PKC)–phosphatidylinositol 3-kinase (PI3K)–Akt pathway. Phosphatase and tensin homologue (PTEN) could inhibit Akt phosphorylation. Upon binding to αv β3, OPN activates AP1 through nuclear factor-inducing kinase (NIK)–ERK (extracellular signal-related kinase) and MEKK1 (mitogen-activated protein kinase kinase kinase1)–JNK1 (c-Jun N-terminal kinase 1) signalling pathways. AP1 promotes cancer cells motility and tumour progression. In addition, transactivation of epidermal growth factor receptor (EGFR) by OPN promotes phosphorylation of ERK which ultimately leads to activation of AP1. Activation of both MMP and HIF-1 pathways leads to enhanced tumour cell survival, proliferation, invasion and angiogenesis^2^

**Fig. 3:**
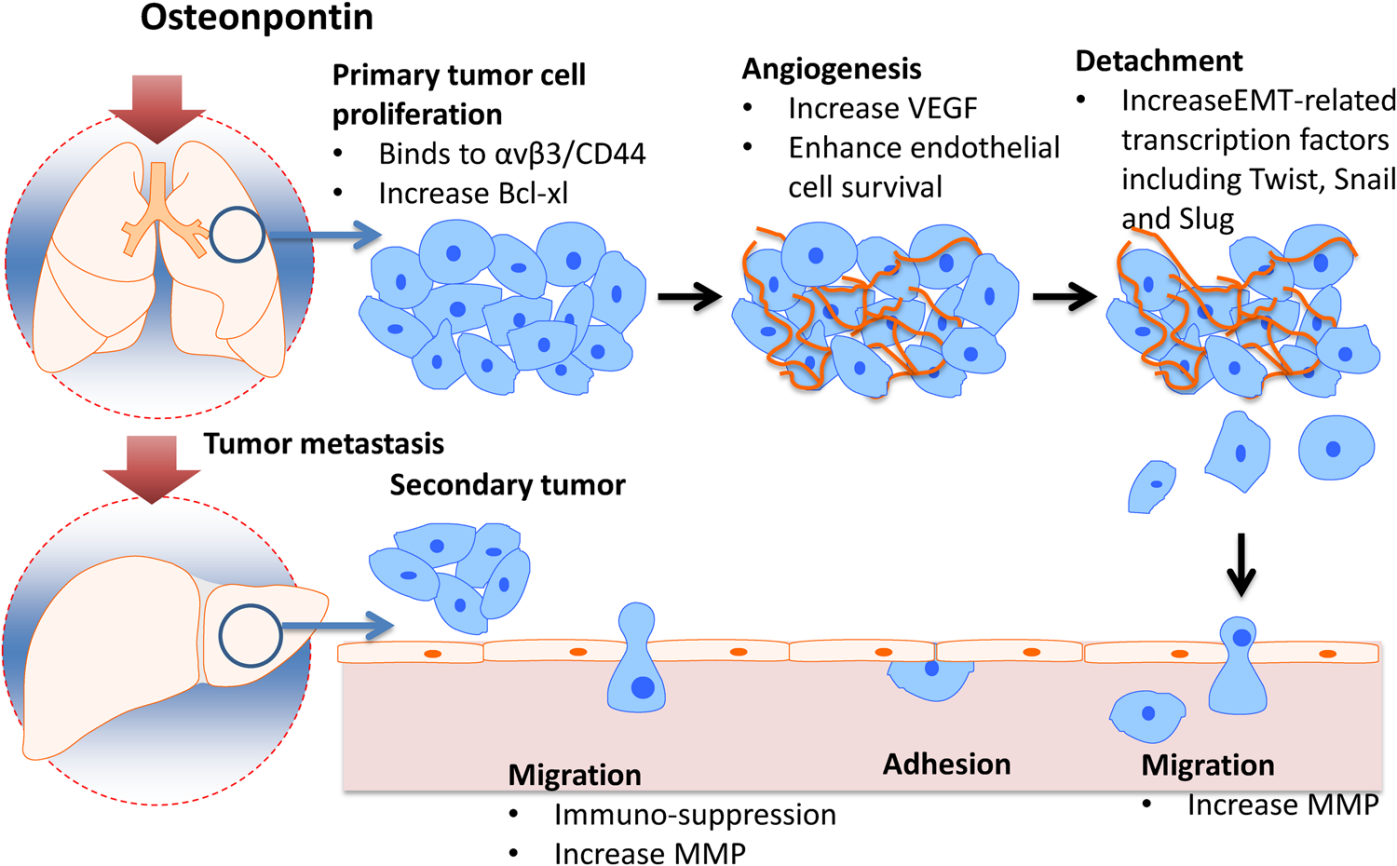
The role of osteopontin in multi-steps of cancer cell metastasis. The OPN overexpression induces multi-steps of cancer cell metastasis through activating different protein mediators. A primary tumour undergoes vascularisation by angiogenesis as various growth factors such vascular endothelial growth factor are secreted. Detachment of the cancerous cell then occurs followed by intravasation; the tumour cell enters and circulates the vascular system. The cell eventually attaches to the wall of the blood vessel before undergoing extravasation and leaving the blood vessel. The tumour cell then grows as a secondary tumour causing metastasis

In addition to its role in mediating normal physiological responses, the role of OPN signalling pathways in cancer progression is becoming increasingly recognised (Fig. 2)^13^ and it has been shown to be involved in multi-steps of tumour metastasis (Fig. 3).

#### Role of osteopontin in tumour cell proliferation

Several studies have demonstrated the correlation between elevated OPN secretion and various malignancies, such as breast and prostate cancer, squamous cell carcinoma, melanoma, osteosarcoma and gliobastoma^1^. The high concentration of OPN cDNA in OPN negative breast cancer cells was shown to promote angiogenesis and, therefore, skeletal metastasis, thus enhancing tumour progression^14^. As well as this, OPN has also been linked to the promotion of tumour cell growth in invasive melanoma, whilst blocking the expression of OPN decreased melanoma cell numbers in vitro. These findings indicate that the recruitment of OPN may be an early feature in the development of melanoma^15^. A study conducted in nude mice demonstrated that NIK is responsible for the stimulation of MMP-9 by OPN and subsequent melanoma growth^1^. Furthermore, it has been found that activation of the melanocyte growth factor receptor stimulates the secretion of OPN, thus encouraging anti-apoptotic signalling and increasing the progression of the melanoma^15^. Overall, these studies suggest that OPN plays a key role in mediating tumour progression by regulating various pathways.

#### Role of osteopontin in tumour angiogenesis

OPN plays a role in the process of angiogenesis due to its high affinity for *α*v*β*3, an integrin highly expressed on particular endothelial cells^1^. Signalling via* α*v*β*3 is essential for endothelial cell survival, and it has been found that OPN promotes the survival of these cells^1^. However, there is no strong evidence to suggest a correlation between OPN and angiogenesis in vivo, and it is more likely that OPN interacts with other pro-angiogenic molecules to stimulate angiogenesis^16^. More studies are required to fully elucidate the role of OPN in mediating angiogenic processes.

#### Role of osteopontin in tumour cell metastasis

Cancer metastasis is a complex process which broadly involves the following processes: the detachment of cancer cells from the primary tumour, intravasation, the circulation of cancer cells, adhesion to the blood vessel wall, extravasation and the growth of the secondary tumour^17^. Although the role of OPN in regulating all of these processes is not completely understood, a meta-analysis published recently reported that overexpression of OPN is closely associated with the metastasis of colorectal cancers, lung cancers and melanomas^18^.

Epithelia-Mesenchymal Transition (EMT) describes the process in which epithelial cells revert back to their mesenchymal phenotype. EMT is characterised by the loss of apical-basal polarity, increased cellular motility and cytoskeleton reorganisation. There are three major types of EMT: type 1 refers to embryogenesis, type 2 is wound healing, and type 3 is cancer metastases. OPN has been shown to play a crucial role in mediating type 2 and 3 EMT^19^. In a breast cancer model, OPN caused an increase in EMT-related transcription factors including, Twist, Snail and Slug^19^. OPN overexpression results in serine phosphorylation of Twist, which then binds to the Bmi-1 promotor, which in turn activates EMT in breast cancer cell lines^20^. OPN is also able to mediate EMT in hepatocellular carcinoma (HCC) models by regulating Twist. In addition, OPN overexpression is shown to activate the PI3K-AKT-Twist pathway, thus promoting EMT and ultimately resulting in HCC metastases^21^. In colorectal cancer, metastasis is also mediated by OPN activating Twist^22^. OPN-mediated Twist activation causes enhanced cell migration, increased invasion and decreased cell-cell adhesion^22^.

In addition, OPN also exerts its function by inducing the hypoxia-inducible factor-1 alpha (HIF-1α) pathway. Intra-tumour hypoxia stabilises HIF-1 alpha, which regulates the expression of Twist by binding to the Twist promotor, thus inducing EMT. OPN is shown to increase HIF-1α via the PI3k/AKT pathway in ovarian cancer and breast cancer models^23, 24^.

OPN has been proposed to shape the tumour microenvironment, thus promoting metastasis in different cancer models. OPN within the tumour milieu can either be tumour-derived or host-derived, and its presence within the tumour milieu may result in enhanced metastasis^25^.

#### Role of osteopontin in tumour chemoresistance

The role of OPN in chemoresistance is currently under investigation, with pre-clinical evidence suggesting that OPN is involved in inducing chemoresistance. Two theories have been proposed to rationalise the association between OPN and chemoresistance.

Autophagy is an evolutionarily conserved catabolic process where organelles are degraded by lysosomes^26^ when cells are subject to cellular stress. A growing body of evidence indicates a paradoxical role of autophagy following chemotherapy, with its response either increasing or reducing chemotherapy anti-cancer activity. Autophagy is shown to promote chemoresistance in some cancer cells during chemotherapy. However, autophagy may also induce autophagic cell death, a form of cell death different to apoptosis^26^. Based on current literature, chemoresistance can be enhanced through the upregulation of autophagy^27–29^. Recent research demonstrated that OPN-induced autophagy via activation of the OPN/NF- κB pathway contributes to chemoresistance to gemcitabine in human pancreatic cancer cells^28^. By silencing OPN expression using lentiviral transfection, gemcitabine conferred enhanced chemotherapy-induced cytotoxic effects in human pancreatic cancer cells^28^. Similar results were obtained from HCC cells^29^. OPN also has chemoresistant effects in HCC cells by activating autophagy via the integrin alpha v beta 3/MEK/ERK1/2 pathway^29^. Lentiviral-mediated miRNA against OPN abolishes the pro-authophagic effect on HCC cells when treated with chemotherapeutic agents^29^.

The second theory proposed to explain the association between OPN and chemoresistance is that OPN may result in anti-apoptotic effects on cancer cells. Yang et al. recently demonstrated that by downregulating OPN expression in breast cancer cell lines with siRNA, the sensitivity of breast cancer cells to doxorubicin was enhanced by increasing cell senescence. The authors concluded that the PI-3K/Akt signalling pathway was potentially responsible for these findings^30^. In addition, another group demonstrated that OPN-knockout breast cancer cells conferred higher levels of cyclophosphamide-induced apoptosis compared to normal breast cancer cells. Furthermore, the authors found that OPN exerted its anti-apoptotic effects on breast cancer cells by activating the p38 MAPK pathway^31^.

### The role of osteopontin on solid organ cancer development

**Fig. 4:**
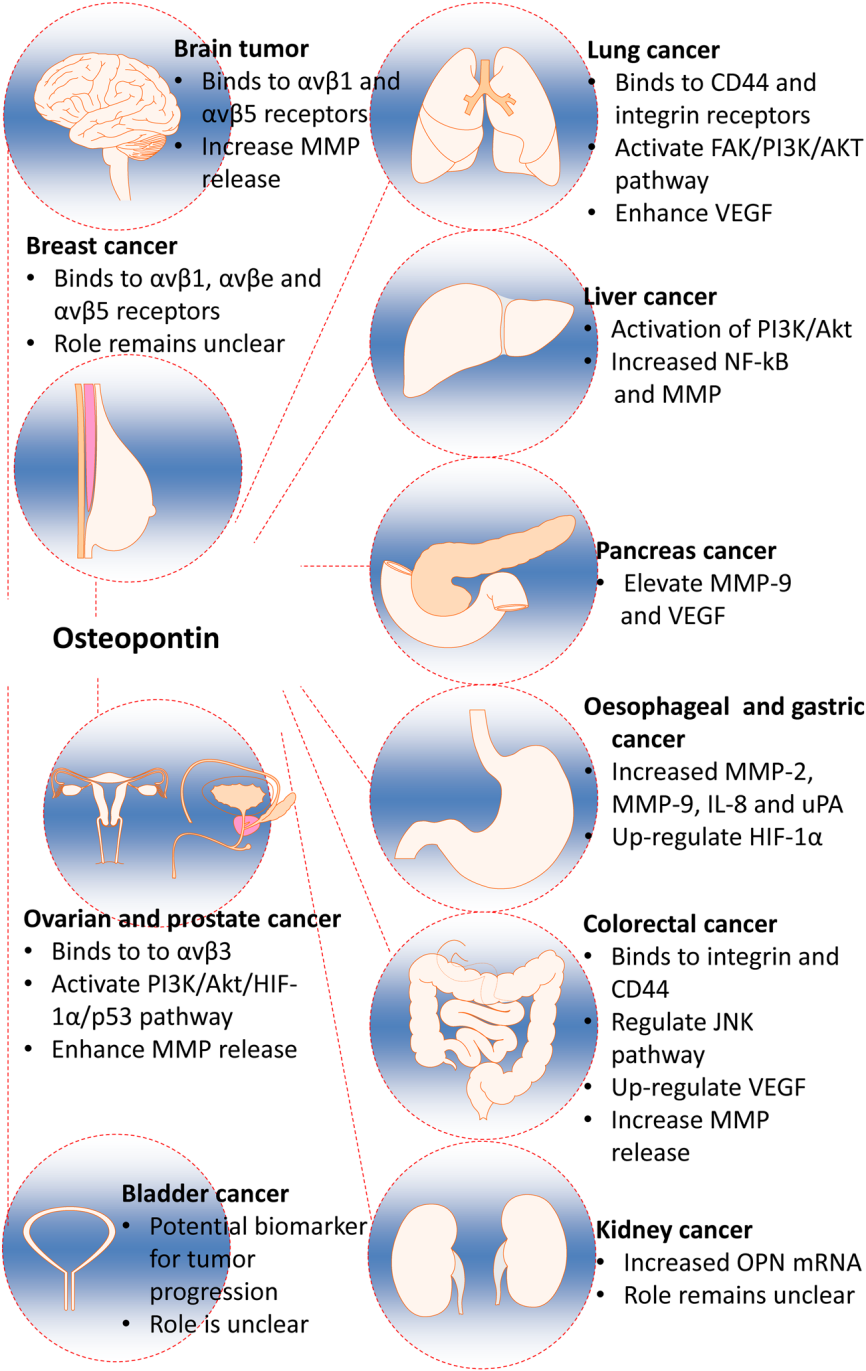
The role of osteopontin in various solid organ tumours. Osteopontin (OPN) has demonstrated a role in the development of various solid organ tumours via various differing mechanisms. In breast, brain, ovarian and prostate cancers, OPN has been shown to preferentially bind to a variety of integrins including αvβ1, and αvβ5, αvβe, αvβ3, resulting in an increase in cell adhesion, migration, and invasion, whilst OPN has been shown to bind to both integrin and CD44 receptors in lung cancers. In addition to receptor binding, OPN is involved in enhancing MMP release and thus increasing cell invasiveness and tumour growth in brain, liver, pancreas, colorectal, ovarian and prostate, and oesophageal and gastric cancers. OPN-mediated upregulation of the PI3K/Akt signalling pathway is a common feature of liver, lung, ovarian and prostate tumour progression, thus preferentially regulating cell survival, cell cycle progression and cellular growth in favour of tumour development. Activation of VEGF and its downstream effector, HIF-1α, by OPN may occur dependently or independently of PI3K/Akt activation and promotes tumour angiogenesis, recruitment of endothelial cells and tumour growth, particularly in colorectal, pancreatic, lung, and oesophageal and gastric malignancies. Activation of the JNK pathway by OPN has been shown to be most specific to colorectal cancer, whilst the precise role of OPN in bladder and kidney cancers, particularly, remains to be elucidated. OPN osteopontin, MMP metalloproteinase, PI3K Phosphatidylinositol-3 kinase, VEGF vascular endothelial growth factor, HIF-1α hypoxia-inducible factor 1

OPN has been shown to play diverse roles in promoting cancer development in different organs (Fig. 4). Recent developments within this area have been summarised as follows:

#### Brain Tumour

Glioblastomas are the most invasive type of glioma and are associated with 5-year survival rates of less than 10%. OPN expression has been associated with increased tumour grade and migratory tendency^32^. Atai et al. (2011) found that in glioblastomas in silico, OPN was one of the 5% most expressed genes in 90% of patients. In situ, the authors also found increased protein levels of OPN in glioblastoma cells compared to normal human brain cells, as well as OPN co-localisation with neutrophils and macrophages. This study suggests that OPN possesses a role in the pathogenesis of glioblastomas, as well demonstrating that OPN in tumours induces the migration of both cancer cells and leucocytes^33^.

Whilst the extracellular matrix of the normal adult brain lacks the expression of adhesion molecules that promote cell attachment, the malignant invasion of astrocytoma tumours is primarily caused by matrix remodelling and upregulation of cellular attachment. Studies have reported the expression of OPN in astrocytic tumours from patient biopsies^4^. It is understood that astrocytomas preferentially express αvβ1, and αvβ5 integrins in vitro and in vivo and are markers of astrocytic malignancy. Ding et al. (2002) demonstrated that OPN’s role in promoting cellular attachment is not limited to non-glial cell types, but that the protein is also involved in promoting malignant astrocytoma cell invasion^34^.

#### Lung cancer

The mechanisms underlying the aggressive phenotype associated with OPN expression have been extensively investigated in non-small-cell lung carcinomas (NSCLC). Firstly, OPN appears to possess a critical role in mediating the process of tumourigenesis. Lin et al. demonstrated that vascular endothelial growth factor (VEGF) and OPN were both overexpressed in NSCLC patient samples and were both significantly associated with clinical features indicating tumour progression^35^. Goutam et al. showed that OPN can induce the accumulation of VEGF via autocrine and paracrine mechanisms, and VEGF subsequently promotes tumourigenesis and development^36^. Secondly, OPN may also play a key role in NSCLC metastasis. EMT is increasingly being recognised as a significant contributor to NSCLC metastasis^19^. OPN can modulate tumour-specific EMT by generating cancer-associated fibroblasts (CAFs)^19^. Furthermore, OPN can induce NSCLC tumour cell migration by interacting with integrins and CD44^37, 38^, and this migration can be blocked by anti-OPN antibodies^38^. OPN may also activate ROCK signalling via the FAK/PI3K/AKT pathway, thus facilitating the invasion of lung cancer cells through lamellipodia formation and the inactivation of cofilin^39^, thereby promoting tumour metastasis. Finally, OPN has been proposed to be a therapeutic target for NSCLC. By delivering PSOT/siOPN complexes to NSCLC cell-xenograft mouse models, Cho et al. recently demonstrated that OPN expression was reduced. In addition, tumour volume and weight were reduced in siOPN-treated groups compared to non-treated group, emphasising the therapeutic potential of OPN^40^.

#### Kidney cancer

A recent review concluded that OPN, together with various other cytokines (e.g IL-8), were promising prognostic biomarkers for progression-free survival (PFS) for patients with renal cell carcinoma (RCC)^41^. The authors selected 50 articles assessing the predictive value of biomarkers using the archived specimens from randomised controlled trails^41^. In fact, Tran et al. reported that IL-8 and OPN were stronger prognostic markers for PFS in the placebo group than standard clinical classifications, such as the Eastern Cooperative Oncology Group performance status, the prognostic models of the Memorial Sloan Kettering Cancer Centre and the International Metastatic Renal Cell Database Consortium^42^. Another study demonstrated that the mRNA level of OPN is a strong indicator of overall survival (OS) and PFS for patients with clear cell renal carcinoma (ccRCC) in both univariate and multivariate analysis^43^. The precise underlying pathways of OPN in RCC are currently obscure and require further investigation.

#### Liver cancer

Hepatocellular carcinoma (HCC) is the most common type of primary liver cancer. Osteopontin is highly expressed in the tumour tissue and serum of patients suffering from many malignancies, including HCC. Studies of surgically resected HCC have demonstrated that osteopontin is highly expressed in tumour tissue and correlates with tumour grade, stage and recurrence^44, 45^. In addition, the expression of osteopontin has also been shown to correlate with the metastatic disposition of HCC^46^. Recent literature also proposes that high serum osteopontin concentrations correlate with poor prognosis, reduced liver function, a worse Child-Pugh score and worse disease-free and overall survival^47–49^. Studies have, therefore, assessed the role of serum osteopontin as a potential tumour biomarker. Evidence exists suggesting that a combination of osteopontin and alpha-fetoprotein enhances the sensitivity and specificity of HCC detection^50^. However, contradictory results have found that osteopontin is relevant in mediating the general hepatic inflammatory environment, rather carcinogenesis specifically^51^. The mechanism by which osteopontin enhances tumour development and metastasis is poorly understood. Huang et al. (2006) provided evidence to suggest that the upregulation of osteopontin expression in hepatitis B-associated HCC may be due to amplification of chromosome 4q21, close to the spp (osteopontin) locus^47^. Furthermore, it has been proposed that osteopontin facilitates tumour progression through activation of downstream signalling, including phosphotidylinositol 3-kinase (PI3K)/Akt, nuclear factor (NF)-kB and matrix metalloproteinases (MMPs)^52^. Sun et al. (2008) also found that osteopontin-mediated activation of mitogen-activated protein kinases (MAPK), NF-kB and MMP-2 pathways was vital in HCC growth and metastases^53^. These findings suggest that osteopontin may provide a novel therapeutic avenue in the treatment of malignancies selectively expressing osteopontin, such as HCC. Bhattacharya et al. (2010) found that miRNA-181a resulted in the notable repression of osteopontin in HCC cell lines, suggesting that epigenetic regulation of osteopontin expression may potentially confer resistance against the metastatic characteristics of HCC^54^.

#### Bladder cancer

Urothelial carcinoma (UC) is the most common type of primary bladder cancer and causes approximately 150,000 deaths annually worldwide^55^. Few studies investigating the correlation between OPN and UC of the bladder have been performed. Findings by Coppola et al. (2004) suggest a correlation between OPN expression and pathological tumour staging in bladder UC, as well as a wide range of other tumour histologies^56^. More recently, Park et al. (2012) conducted a prospective study to determine the value of plasma OPN levels as a predictive factor of disease stage and recurrence in patients with bladder UC^57^. The prominent findings of the study were the correlation between preoperative plasma OPN levels and muscle invasion, as well as an increase in plasma OPN with disease burden in bladder UC. These results suggest that OPN may be a potential marker for predicting risk and clinical prognosis in patients with bladder UC. Findings by Ke et al. (2011) indicate that OPN may also be a potential biomarker for other urinary tract urothelial carcinomas, including UC of the renal pelvis and ureters^58^.

#### Breast cancer

The relationship between OPN and breast cancer progression was initially studied by Tuck et al. (1997) by extracting tissue samples from a patient with synchronous, bilateral, invasive mammary carcinomas of the same histology that later developed metastatic recurrence^59^. Subsequently, the same authors investigated OPN protein and mRNA expression in the tumours of 154 women with lymph-node negative breast cancer^60^. OPN was identified in tumour infiltrating macrophages and lymphocytes in 70% of tumours and also localised specifically to carcinoma cells in 26% of tumours. Overall, the results of this pilot study suggest that tumour aggressiveness and poor prognosis is associated with the ability of breast cancer cells to either synthesise OPN or to bind and sequester OPN from the tumour^61^ microenvironment. More recently, the potential for OPN to provide diagnostic, prognostic and clinical information for patients with breast cancer has been demonstrated. Serum levels of OPN are increased by up to 10-fold in patients with disseminated breast cancer, with higher concentrations associated with higher tumour grade^62–64^. A recent study investigating integrin-mediated induction of cellular migration found that the αvβ3 integrin is intrinsically involved in the migration of a highly tumorigenic, metastatic breast cancer cell line towards OPN, whilst non-metastatic cells were found to use αvβ1, and αvβ5 integrins^65^. Various other studies have supported the notion that tumour cells capable of binding αvβ3 and OPN possess more aggressive tendencies and a significant survival advantage^66^.

#### Oesophageal cancer

Studies have found that higher expression of OPN, including all its isoforms, OPNa, OPNb, OPNc, is seen in oesophageal cancer^67^. The overexpression of different isoforms confers different cancer cell characteristics that promote tumorigenesis. For instance, OPNb promotes cell proliferation and migration, but increased cell adhesion. In contrast, OPNc prevents cell migration but promotes cell detachment. OPN may be used as a prognostic marker in oesophageal cancer. One study demonstrated that more advanced oesophageal cancer is associated with higher expression of OPN, although the higher level of OPN cannot predict patient survival^67^. Another study, however, demonstrated that patients with oesophageal cancer that express more OPN had poorer survival rates^68^. The inconsistency between these studies indicate the necessity for further research to elucidate the role of OPN in the prognostic prediction of oesophageal cancer.

#### Pancreatic cancer

An increased serum level of OPN has been seen in pancreatic cancer patients ^69^and in vitro experiments have demonstrated that OPN mRNA is upregulated in pancreatic cancer cell lines^69^. Using anti-sense oligonucleotides to downregulate OPN demonstrates that it is possible to inhibit cell proliferation^70^. Metastasis is seen frequently in pancreatic cancer patients. The increased expression of OPN mRNA has been seen in mice liver metastatic cell lines (HPC-3H4) that are derived from pancreatic cancer cell lines^71^. In smoking patients, metastasis of pancreatic cancer cells seems to be associated with the upregulation of OPN. Lazar et al. demonstrated that OPN plays a role in elevating the levels of MMP-9 and VEGF, both of which are important in tumour development and metastasis. The increase in MMP-9 and VEGF is attenuated by inhibiting the expression of OPN^72^. Hence, OPN may be a therapeutic target for pancreatic cancer.

Since there has been an established association between an elevated level of OPN and pancreatic cancer, it has been suggested that OPN may be used as a biomarker^73^. Poruk et al. demonstrated that tissue inhibitor of metalloproteinase 1 (TIMP-1) is elevated in pancreatic cancer, but not chronic pancreatitis and, therefore, the combination of TIMP-1 and OPN may help differentiate between the two^74^.

#### Gastric cancer

It has been established that Helicobacter Pylori (H. pylori) infection is one of the risk factors for gastric cancer^75^. An in vivo study involving OPN knockout (KO) mice showed that OPN plays a vital role in the development of gastric cancer from H. Pylori infection^76^. The study demonstrated that the size of gastric tumours is larger and the incidence of gastric cancer is higher in wild-type (WT) mice expressing OPN, compared with OPN KO mice. It was observed that the loss of OPN is associated with increased levels of inducible nitric oxide synthase (iNOS) and its regulator, signal transducer and activator of transcription 1 (STAT1), both of which have indicated the ability to induce apoptosis and growth arrest^76–79^.

OPN may also possess anti-apoptotic properties in the tumourigenesis of gastric cancer by modulating the balance between pro-apoptotic and anti-apoptotic factors. It has been observed that increased levels of OPNb and OPNc are associated with an increase in anti-apoptotic Bcl-2, and a decreased production of pro-apoptotic capase-3 and Bax, hence facilitating tumour survival^80^. In addition to its anti-apoptotic properties, OPN also appears to promote invasion and metastasis in gastric cancer. Overexpression of OPN can cause an increased expression of MMP-2, MMP-9, IL-8 and urokinase plasminogen activator (uPA), as well as inhibition of caspase 3 generation, thus promoting cancer cell invasion and metastasis^80, 81^. Furthermore, OPN may also promote angiogenesis and tumour growth in gastric cancer by upregulating VEGF^82^. Although the mechanism for this remains to be fully elucidated, it is believed that OPN promotes the translocation of NF-Κb via the MAPK and PI3K/Akt pathways, which upregulates HIF-1α to promote cancer cell proliferation and survival^81, 83^.

#### Colorectal cancer

Although the precise mechanisms underlying OPN’s ability to promote tumourigenesis are not fully understood, studies have demonstrated that OPN may bind to CD44v6 to promote colorectal cancer cell proliferation and survival, possibly via the JNK pathway^84^. It is also evident that OPN may promote the expression of cancer stem cell markers, such as OCT4 and SOX2, which not only improves cancer cell survival but also enhances chemotherapeutic resistance, namely to oxaliplatin^85^.

OPN may be an alternative prognostic marker in colorectal cancer. Multiple studies have indicated that high expression of OPN is associated with lymph node metastasis, postoperative metastasis, venous invasion, advanced staging and poorer survival^22, 86–88^. A recent meta-analysis of 15 studies has demonstrated that overexpression of OPN correlates with more advanced tumour grade, lymph node and distant metastasis, and poorer 2-year, 3-year and 5-year survival rates. However, no significant correlation has been found between increased OPN and depth of tumour invasion^89^.

#### Prostate cancer

Evidence has shown that OPN is closely associated with the proliferation and metastasis of prostate cancer^90, 91^. A study involving genetically engineered mice demonstrated that the expression of OPN occurs at the early stages of prostate neoplasm development, and a high level of OPN is observed in developed adenocarcinomas and metastatic deposits^90^. The molecular mechanisms underlying OPN-mediated tumourigenesis in prostate cancer has been explored in several studies. It appears that the binding of OPN to integrin αvβ3 may activate multiple signalling cascades, thus promoting tumourigenesis. For instance, OPN, upon binding to αvβ3, can activate Rho GTPase via RANKL, subsequently upregulating the expression of CD44 and MMP-9 and resulting in the increased ability for cancer cell movement and metastasis^92^. It is also possible that the binding of OPN to αvβ3 may promote the formation of invadopodia via the WASP-Arp2/3 pathway and upregulation of VEGF via the MAPK pathway, which also results in enhanced prostate cancer cell invasion^93, 94^. Furthermore, OPN may have an important regulatory role in the activation of ERK1/2 in prostate cancer. It has been shown that OPN can phosphorylate c-Raf to activate the ERK1/2, but also indirectly inhibits it by activating Akt^95^. Additionally, the binding of OPN to αvβ3 also appears to activate the PKCα/c-Src/IKK/NF-kB signalling cascade, resulting in the upregulation of COX-2 expression and PGE2 production, which further promotes cancer cell invasion and angiogenesis^96^. In addition to binding to αvβ3, an in vitro study further speculated that OPN may also bind to integrin β1 to maintain the activation of EGFR, which allows the proliferation of tumour cells^97^.

#### Ovarian cancer

Many studies have been conducted to evaluate the correlation between increased levels of OPN and ovarian cancer. In a meta-analysis of 15 studies, it was found that higher serum levels of OPN were positively associated with ovarian cancer and may be a risk factor in Asian populations^98^. It was discovered that among the three isoforms, OPNa, OPNb and OPNc, OPNc is the only isoform that is overexpressed in ovarian tumours^99^. Furthermore, OPNc is thought to be associated with increased tumour cell proliferation, migration and invasion^99^. In the human ovarian cancer cell line, OvCar-3, overexpression of OPNc has been shown to be associated with the upregulation of multiple genes. These are responsible for angiogenesis, cell cycle control, metastasis and cell adhesion^100^. It is thought that OPNc may promote these features of tumour development by upregulating HIF-1α via the activation of the PI3K/Akt/HIF-1α/p53 signalling pathway^23^.

Clear cell carcinoma of the ovary (CCCO) is a subtype of ovarian epithelial carcinoma and is associated with a higher resistance to platinum-based chemotherapy, thus conferring a poorer prognosis^101^. Overexpression of OPN has also been observed in CCCO^102, 103^. It is speculated that HNF-1β, a transcription factor, plays a role in the upregulation of OPN in CCCO, and that OPN promotes invasion by binding to several integrins, including αvβ1, αvβ3, αvβ5 and α5β1^102, 103^. Interestingly, Matsuura et al. demonstrated that the administration of simvastatin downregulates the expression of OPN and integrins, both of which reduce extracellular matrix invasion^102^. Although the mechanism of this reduction is not fully understood, it is thought that statins may deplete OPN expression by inhibiting HMG-CoA reductase and reducing isoprenoids^104^.

### Osteopontin as therapeutic target

It has been suggested that OPN produced from tumours lacks important domains and is, therefore, structurally different to other types of OPN. As a result, blocking its production could be used to reduce tumour progression^105^. There are currently two methods to reduce OPN gene expression on an RNA level: ribozyme cleavage and hybridisation with antisense oligonucleotides. However, both methods are limited due to the difficulty in delivering nucleic acid drugs on an intracellular level^106^. Inhibiting OPN protein has been attempted using antibodies or synthetic peptides^105^. Several antibodies have been developed that identify specific epitopes of OPN^107^. Polyclonal antibodies to OPN prevent increased tumour growth in human prostate carcinoma cells, whilst murine anti-human OPN antibody has been shown to prevent the adhesion of MDA-MB-435 breast cancer cells^105^. As inhibiting adhesion of epithelial cells is linked to increased apoptosis, it may be possible that preventing cancer cell adhesion to OPN may also stimulate apoptosis of tumour cells, further ameliorating cancer treatment. A list of potential therapeutic strategies presented in Table 1

**Table 1:** Therapeutic strategy targeting osteopontin in cancer

Targeting strategy	Inhibitors	Target molecules (s)	Targeting cancer/tissue	Effect/outcome
RNAi	siRNA^117^	OPN, CD44	Breast cancer	OPN silencing causes reduced growth, migration and invasion. CD44 silencing abolishes OPN induced signalling
	siRNA^118^	OPN	Mammary cancer	Impairs cancer cells proliferation, survival and migration
	siRNA^119^	OPN	Breast cancer	Inhibits migration and invasion. Knockdown affects PI3K/Akt/mTOR pathway and promotes expression of autophagy-related gene products LC3 and Beclin 1
	siRNA^40^	OPN	Non-small lung cancer	siRNA was delivered by polysorbitol-based transporter (POST). POST-delivered animals demonstrated reduced tumour volume and weight.
	miRNA^54^	OPN	Hepatocellular carcinoma	Reduced metastatic potential in HCC
Aptamers	OPN-R3^116^	OPN	Brest cancer	Decreases cellular adhesion, migration and invasion in breast cancer cells.
Small-molecule inhibitors	Parecoxib^120^	NR4A2 and Wnt Signalling	Intestinal polyps	Reduces disease burden
	Simvastatin^102^	3-Hydroxy-3-methylgluaryl coenzyme A reductase	Ovarian cancer	Induces apoptosis and cell growth arrest by reducing OPN expression
	Andrographolide^115^	OPN	Breast cancer	Reduces cancer growth
	Trichostatin A^121^	HDAC inhibitor	Cervical cancer	Inhibits PMA-induced tumour growth
	Curcumin + BPs^122^	OPN, CD44 and MMP-9	Ovarian cancer	Reduces invasions of ovarian cancer and dendritic cells
	Agelastatin A^123^	b-Catenin and Tcf-4 signalling	Breast cancer	Inhibits cell invasion and adhesion
Blocking antibody	Anti-OPN Antibody^124^	OPN	Lung and breast cancer	Inhibits cell migration adhesion, invasion and metastatic potential of cancer cells
	Anti-OPN antibody^118^	OPN	Mammary cancer	Impairs cancer cells proliferation, survival and migration
	Anti-αv3β integrin antibody^125^	αvβ3-integrin	Breast cancer	Inhibits OPN-induced tumour growth and angiogenesis
	Anti-CD44 antibody^126^	CD44 receptor	Fibroblasts	Reduces tumour progression induced by OPN

As evidenced above, OPN is a promising therapeutic target in the management of various malignancies in the future, due to its role in enhancing tumour-mediated necrosis, cell proliferation, survival, invasion, angiogenesis, metastatic potential and drug resistance^25, 108^. Various pre-clinical studies have recently investigated the role of OPN inhibition in managing metastatic disease. For instance, Bandopadhyay et al. produced a review indicating the ability for small interfering RNA and short hairpin RNA against OPN to inhibit tumour progression and metastasis^109^. In addition, Wu et al. demonstrated that OPN knockout mice experienced slower tumour growth, whilst OPN knockout B16 melanoma cells had a reduction in metastasis to bone and soft tissues^110, 111^. Furthermore, Wu conducted a study investigating the effects of OPN knockout in colon cancer cells using four siRNA molecules of a target OPN gene^112^. The team’s findings indicate that OPN silencing results in the inhibition of various downstream effector cascades, involving a reduced expression of urokinase plasminogen activator, VEGF, MMP-2 and MMP-9, which in turn may clinically translate into a reduction in colon cancer invasion, angiogenesis and metastasis. In addition, attenuation of OPN expression is associated with downregulation of HIF-1 and VEGF in MDA-MB-343 breast cancer cells, whilst stable OPN-silencing results in a reduction in cell invasion, an increase in cell apoptosis and senescence, as well as a reduction in clonogenic survival^113^. Together, these findings indicate that OPN silencing results in enhanced radiosensitivity and mediation of cellular apoptosis in breast cancer cells.

The use of blocking antibodies to directly target OPN and its receptors, CD44 and α_v_β_3_-integrin, has also demonstrated promising results. It has been shown that targeting CD44 on mesenchymal stromal cells results in a reduction in OPN-mediated tumour growth, whilst blocking antibodies to α_v_β_3_-integrin and targeting of 4T1 cell-surface integrins in murine mammary epithelial cancer cells are both associated with an attenuation of ILK, MMP-2 and uroplasminogen activator expression^114^.

Small molecule inhibitors have also been shown to attenuate OPN expression. For instance, Andrographolide, a diterpenoid lactone sequestered from *Andrographis paniculata*, inhibits breast cancer cell proliferation via the attenuation of the PI3kinase/Akt signalling pathway^115^.

Finally, aptamers have been classically considered as promising therapeutic options, primarily due to their biological stability, capacity for immunogenic resistance and low-dose efficacy. A study designed to characterise the critical sequence of an RNA aptamer, OPN-R3, directed against human OPN in MDA-MB231 human breast cancer cells^116^ found that exposure to OPN-R3 was associated with significant downregulation of OPN signal transduction pathways, PI3K, JNK1/2, Src and Akt, as well as extracellular matrix degradation pathways involving matrix metalloproteinases. An in vitro model found that OPN-R3 inhibits MDA-MB231 adhesion by 60%, migration by 50% and invasion by 65%, whilst an in vivo xenograft model of breast cancer indicated that the aptamer significantly decreased local progression and distant metastases^116^.

## Conclusion

There is well-documented evidence to suggest that OPN contributes to tumour progression. However, further research is necessary to improve the current understanding of these various molecular pathways and elucidate the precise role of OPN in mediating solid cancer progression and metastasis. OPN has the potential to be a novel biomarker and anti-cancer therapeutic target to manage tumour progression in various malignancies, thus heralding the possibility for earlier detection and treatment of cancer and metastasis.
